# Supplementary material for: Higher Ultra-Processed Food Consumption Is Associated with Greater High-Sensitivity C-Reactive Protein Concentration in Adults: Cross-Sectional Results from the Melbourne Collaborative Cohort Study
Source: Nutrients. 2022 Aug 12;14(16):3309. doi: 10.3390/nu14163309 (PMC9415636; doi:10.3390/nu14163309)
Supplement: Supplementary file 1 [file nutrients-14-03309-s001.zip › nutrients-1842047-supplementary.pdf]

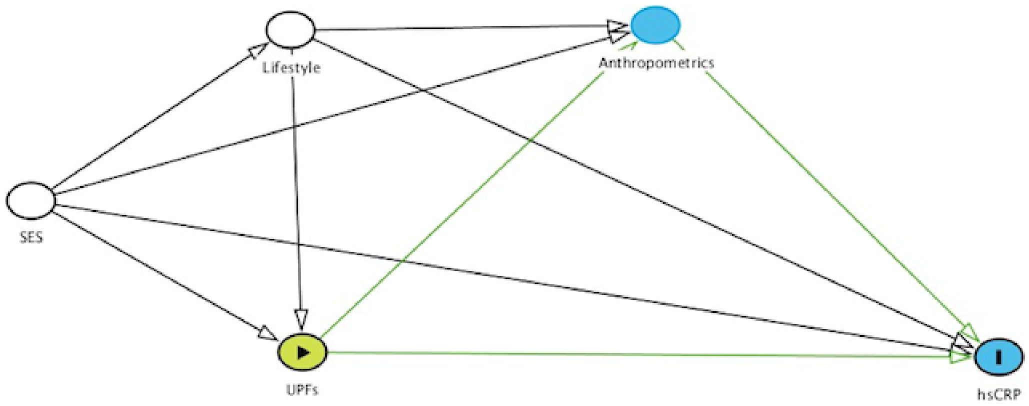

Figure S1. A directed acyclic graph mapping hypothesised relationships between all relevant variables. SES, socio-economic status; hsCRP, high-sensitivity C-reactive protein; UPFs, ultra-processed foods.

Table S1. Sensitivity excluding individuals with hsCRP concentrations above 10 mg/L and cardiovascular disease mortality.

**Excluding individuals with hsCRP concentrations above 10 mg/L**			
	n	**Estimated relative change in hsCRP concentration (mg/L) for each energy-adjusted 100 (g) increase in ultra-processed food intake (95%CIs)**	***p*-value**
Model 1 [a]	1896	3.5% (1.6%—5.4%)	<0.001
Model 2 [b]	1785	3.6% (1.7%—5.5%)	<0.001
*Model 3 [c]	1739	3.6% (1.7%—5.5%)	<0.001
**Model 4 [d]	1737	2.6% (0.8%—4.4%)	0.004
**Excluding individuals with cardiovascular disease mortality**			
	n	**Estimated relative change in hsCRP concentration (mg/L) for each energy-adjusted 100 (g) increase in ultra-processed food intake (95%CIs)**	***p*-value**
Model 1 [a]	1451	3.5% (0.9%—6.0%)	0.009
Model 2 [b]	1382	4.3% (1.8%—6.9%)	0.001
*Model 3 [c]	1361	4.1% (1.6%—6.6%)	0.001
**Model 4 [d]	1426	2.4% (0.0%—4.7%)	0.046

[a] Model 1 = unadjusted. [b] Model 2 = additionally adjusted for sociodemographic characteristics: sex (male, female), age (continuous), education ((in)completed tertiary degree or diploma, completed high/technical school, (in)completed high/technical school, completed primary school, (in)completed primary school), country of birth (Australia/New Zealand/Other, United Kingdom/Malta, Italy, Greece), marital status (married, de facto, divorced, separated, widow) and SEIFA quintiles (Q1–Q5). [c] *Model 3 = main model additionally adjusted for lifestyle and health related behaviours: smoking status (never smoked, current smoker, former smoker), physical activity over the last 6 months (0 (none), >0 and <4 (low), ≥4 and <6 (moderate), ≥6 (high)), and alcohol intake (g/day) (lifetime abstainers, ex-drinkers, up to 19, 20–29, 30–39, 40+). Change to *n* due missing values for confounder alcohol intake. [d] **Model 4 = supplementary analyses additionally adjusted for body mass index (kg/m^2). Change to *n* due missing values for confounders alcohol intake and body mass index. hsCRP, high-sensitivity C-reactive protein; CIs, confidence intervals; SEIFA, Socio-Economic Indexes for Areas.

Table S2. Sensitivity analyses excluding individuals with history of non-communicable diseases.

	n	Estimated relative change in hsCRP concentration (mg/L) for each energy-adjusted 100 (g) increase in ultra-processed food intake (95%CIs)	*p*-value
Hypertension	1352	3.9% (1.5%—6.3%)	0.002
Stroke	1813	3.6% (1.5%—5.8%)	0.001
Heart attack	1733	3.8% (1.7%—5.9%)	<0.001
Diabetes	1763	3.6% (1.4%—5.7%)	0.002
Cancer	1699	3.8% (1.7%—5.9%)	<0.001
Body mass index ≥30	1383	2.3% (−0.1%—4.8%)	0.063

Main model adjusted for: sex (male, female), age (continuous), education ((in)completed tertiary degree or diploma, completed high/technical school, (in)completed high/technical school, completed primary school, (in)completed primary school), country of birth (Australia/New Zealand/Other, United Kingdom/Malta, Italy, Greece), marital status (married, de facto, divorced, separated, widow), SEIFA quintiles (Q1–Q5), smoking status (never smoked, current smoker, former smoker), physical activity over the last 6 months (0 (none), >0 and <4 (low), ≥4 and <6 (moderate), ≥6 (high)), and alcohol intake (g/day) (lifetime abstainers, ex-drinkers, up to 19, 20–29, 30–39, 40+).

Table S3. Sex-stratified cross-sectional associations between the ultra-processed food intake and hsCRP concentration (MCCS, 1990–1994).

Variable	*n*	Estimated relative change in hsCRP concentration (mg/L) for each energy-adjusted 100 (g) increase in ultra-processed food intake (95%CIs)	*p*-value
**Men**			
Model 1 [a]	1261	3.4% (1.1%—5.6%)	0.003
Model 2 [b]	1185	3.4% (1.2%—5.6%)	0.003
*Model 3 [c]	1148	3.5% (1.3%—5.7%)	0.002
**Model 4 [d]	1148	2.8% (0.7%—4.9%)	0.010
**Women**			
	n	**Estimated relative change in hsCRP concentration (mg/L) for each energy-adjusted 100 (g) increase in ultra-processed food intake (95%CIs)**	***p*-value**
Model 1 [a]	757	5.8% (1.0%—10.6%)	0.018
Model 2 [b]	714	5.8% (0.8%—10.7%)	0.023
*Model 3 [c]	704	5.5% (0.5%—10.5%)	0.032
**Model 4 [d]	702	2.4% (−2.1%—6.8%)	0.296

[a] Model 1 = unadjusted. [b] Model 2 = additionally adjusted for sociodemographic characteristics: sex (male, female), age (continuous), education ((in)completed tertiary degree or diploma, completed high/technical school, (in)completed high/technical school, completed primary school, (in)completed primary school), country of birth (Australia/New Zealand/Other, United Kingdom/Malta, Italy, Greece), marital status (married, de facto, divorced, separated, widow) and SEIFA quintiles (Q1–Q5). Change to *n* due missing values for confounders marital status and SEIFA quintiles. [c] *Model 3 = main model additionally adjusted for lifestyle and health related behaviours: smoking status (never smoked, current smoker, former smoker), physical activity over the last 6 months (0 (none), >0 and <4 (low), ≥4 and <6 (moderate), ≥6 (high)), and alcohol intake (g/day) (lifetime abstainers, ex-drinkers, up to 19, 20–29, 30–39, 40+). Change to *n* due missing values for confounder alcohol intake. [d] **Model 4 = supplementary analyses additionally adjusted for body mass index (kg/m^2). Change to *n* due missing values for confounders alcohol intake and body mass index. MCCS, Melbourne Collaborative Cohort Study.
